# Overexpression of TFF3 is Associated with Immune Infiltration, Molecular Subtypes, and Clinical Progression in Breast Cancer

**DOI:** 10.1007/s11596-026-00173-0

**Published:** 2026-03-04

**Authors:** Bei Liu, Qin Wang, Rong-fu Huang, Xiao-hong Min, Han-han Liu, Huan Wu, Hui Xu, Jun-bo Hu, Yong-qing Tong, Zi-ming Huang

**Affiliations:** 1 Department of Pathology, Maternal and Child Health Hospital of Hubei Province, Wuhan, 430070 China; 2 Department of Clinical Laboratory, Second Affiliated Hospital of Fujian Medical University, Quanzhou, 362000 China; 3 Prenatal Diagnostic Center of Maternal and Child Health Hospital of Hubei Province, Wuhan, 430070 China; 4 Department of Breast and Thyroid Surgery, Maternal and Child Health Hospital of Hubei Province, Wuhan, 430070 China

**Keywords:** TFF3, Breast cancer, Immune microenvironment, Prognostic biomarker, Molecular subtyping

## Abstract

**Objective:**

Trefoil factor 3 (TFF3), a secreted protein involved in mucosal protection and tumor progression, has an incompletely defined role in breast cancer (BRCA). This study aimed to comprehensively evaluate TFF3 expression patterns, clinical relevance, and prognostic significance in BRCA.

**Methods:**

Data from the TCGA, GTEx, TNMplot, and TISCH2 databases were integrated to analyze TFF3 expression and clinical significance. Protein expression in clinical samples was validated via immunohistochemistry (IHC), and survival analysis, immune infiltration assessment, and functional enrichment analyses were performed to explore the biological role of TFF3.

**Results:**

TFF3 was significantly upregulated in BRCA tumor tissues compared with normal tissues (*P* < 0.001) and was expressed predominantly in malignant cells and tumor-associated macrophages. High TFF3 expression correlated strongly with hormone receptor (estrogen receptor/progesterone receptor) positivity, luminal A/B subtypes, and early-stage disease (*P* < 0.01) and showed excellent diagnostic performance for distinguishing basal-like from non-basal-like BRCA (AUC = 0.95). TFF3 was an independent protective factor: high expression was associated with improved overall survival (HR = 0.75, *P* = 0.02) and disease-free survival (*P* < 0.001), especially in patients receiving hormone therapy (HR = 0.89, *P* = 0.0002) or chemotherapy (HR = 0.89, *P* = 0.0002). TFF3 exhibited immunomodulatory properties, correlated positively with M2 macrophages and negatively with cytotoxic immune cells (CD8^+^ T cells, NK cells) and checkpoint molecules (PD-1, CTLA-4) (*P* < 0.01). Functional analyses linked TFF3 to estrogen response pathways and cell cycle regulation. IHC validation confirmed TFF3 overexpression in 78.1% of tumors versus 23.9% of normal tissues (*P* < 0.001), with the lowest expression in the basal-like subtype (8.33% vs. 59.2%, *P* = 0.0007).

**Conclusions:**

TFF3 is a robust diagnostic biomarker for BRCA molecular subtyping, an independent prognostic factor, and a potential immunomodulator. These findings highlight its clinical utility for patient stratification and potential as a therapeutic target, particularly in hormone receptor-positive BRCA.

## Introduction

Breast cancer (BRCA) is one of the most prevalent malignancies worldwide. In 2020, an estimated 2.3 million new cases (11.7% of all cancers) were diagnosed worldwide, establishing that BRCA is the most commonly diagnosed cancer, surpassing the incidence of lung cancer. In the same year, 685,000 BRCA-related deaths were recorded, making it the fifth leading cause of cancer mortality [1]. Notably, epidemiological data reveal a rising trend in both the incidence and mortality of BRCA in China, with 416,371 new cases and 117,174 deaths reported in 2020 [2]. BRCA encompasses a heterogeneous group of diseases with diverse pathological features and clinical prognoses. Current classification systems stratify BRCA into five molecular subtypes on the basis of histopathological markers (hormone receptor status) and proliferation indices (Ki-67 levels): HER2-enriched, triple-negative, luminal A-like, luminal B-like HER2-negative, and luminal B-like HER2-positive [3]. Standard therapeutic approaches include surgery, chemotherapy, and radiotherapy [4]. Advances in these modalities have contributed to a 40% reduction in BRCA mortality between 1979 and 2017 [5]. Nevertheless, approximately 10% of patients develop chemotherapy-resistant metastases or inoperable disease, leading to poor outcomes [6]. Emerging immunotherapies, which target the tumor microenvironment (TME), represent a promising therapeutic paradigm for overcoming treatment resistance.

The TME consists of immune cells, stromal cells, tumor cells, and secreted cytokines/chemokines, which collectively regulate tumor cell physiology [7]. Immune checkpoints play a vital role in preventing excessive immune activation; however, tumors often hijack these pathways to evade immune surveillance. Blockade of immune checkpoints can remodel the TME and counteract tumor immune escape. The clinical success of immune checkpoint inhibitors (ICIs) and chimeric antigen receptor (CAR) T-cell therapy across multiple cancers led to the distinction of *Science*’s 2013 “Breakthrough of the Year” [8]. Among immune checkpoints, programmed death-ligand 1 (PD-L1) is the most widely studied. It is expressed predominantly on tumor cells and a subset of tumor-infiltrating lymphocytes (TILs). In BRCA, PD-L1 is detected in approximately 5% of triple-negative BRCA (TNBC) cases. While PD-L1 inhibitors combined with chemotherapy show modest efficacy in advanced/metastatic TNBC [9], primary or acquired resistance to immunotherapy remains a major limitation [10]. Moreover, PD-L1 expression is significantly lower in non-TNBC subtypes than in TNBC subtypes [9]. Consequently, discovering novel immune checkpoints for BRCA immunotherapy is critically important.

Previous studies have demonstrated that trefoil factor 3 (TFF3), a secreted peptide belonging to the trefoil factor family, plays a multifaceted role in tumor progression and metastasis through its interactions with mucins and growth factor receptors [11]. Among the trefoil factors, TFF3 is particularly noteworthy because of its unique structural characteristics and dual role in both mucosal protection and oncogenic processes [12]. TFF3 contains a distinctive trefoil domain composed of three conserved disulfide bonds, enabling its resistance to proteolytic degradation in the TME [13]. Its involvement in cancer pathogenesis has been increasingly recognized [14].

TFF3 is overexpressed in numerous human carcinomas, including breast, colorectal, and prostate cancers, and its elevated expression is significantly correlated with advanced tumor stage and poor prognosis [15]. Targeting TFF3 may represent a promising therapeutic strategy, especially for hormone receptor-positive tumors, as TFF3 expression has been shown to promote estrogen receptor (ER) signaling while being independent of HER2 amplification in BRCA [16]. In the TME, TFF3 secreted by cancer-associated fibroblasts (CAFs) enhances tumor cell proliferation and inhibits apoptosis through activation of the PI3K/AKT and ERK pathways [17].

The pro-tumorigenic effects of TFF3 have been validated both in vivo and in vitro, where it was found to stimulate epithelial‒mesenchymal transition (EMT) and facilitate metastatic dissemination [18]. Mechanistic studies revealed that TFF3 binds to CXCR4 chemokine receptors on tumor cells, triggering downstream signaling cascades that upregulate matrix metalloproteinases (MMPs) and promote extracellular matrix (ECM) remodeling [19]. In clinical cohorts of patients with metastatic colorectal cancer, high serum TFF3 levels are associated with resistance to anti-EGFR therapy [20]. Furthermore, TFF3 overexpression has been clinically documented in gastric cancer and hepatocellular carcinoma, where it serves as an independent predictor of lymph node metastasis [21, 22].

In this study, we systematically analyzed TFF3 expression and its clinical significance in BRCA via multiomics data from The Cancer Genome Atlas (TCGA), Genotype-Tissue Expression (GTEx), and University of California Santa Cruz (UCSC) Xena databases, complemented by survival analysis via the Kaplan‒Meier (KM) plotter. We further validated TFF3 protein expression patterns and their prognostic value through immunohistochemistry (IHC) in BRCA samples. Additionally, we performed gene enrichment analysis to elucidate TFF3-related pathways and assessed their impact on tumor immune infiltration. To strengthen the clinical relevance of TFF3, we evaluated TFF3 protein expression in 149 human BRCA tissue samples and correlated its levels with clinicopathological parameters and overall survival (OS) outcomes.

## Materials and Methods

### Data Processing and Differential Expression, Survival, and Correlation Analysis

We utilized the TCGA dataset and the Genotype-Tissue Expression (GTEx) database to collect raw RNA-sequencing data and accompanying clinical information for tumor tissues and adjacent tissues from BRCA [23]. All the analyses were conducted via R software, version 3.6.3. The R packages “ggplot2”, “survminer”, and “survival” were employed to plot expression analysis and KM survival curves. Univariate Cox proportional hazards regression was used to calculate *P* values and hazard ratios (HRs) with 95% confidence intervals (CIs) in the KM curves. Log-rank tests were used to determine statistical significance. The R package “ggstatsplot” was used for the two-gene correlation analysis. Pearson’s correlation or Spearman’s correlation analysis was used to assess the correlation between quantitative variables. The R package “pROC” was used to analyze the diagnostic significance of TFF3 mRNA. The Single-sample Gene Set Enrichment Analysis (ssGSEA) algorithm provided in the R package “GSVA” was used to calculate immune infiltration levels via markers of 24 immune cell types.

### UCSC Xena

We downloaded RNA-seq data in TPM format for TCGA and GTEx, which were uniformly processed via the Toil process from UCSC Xena (https://xenabrowser.net/datapages/), allowing us to view and analyze public and private, multiomic and clinical/phenotypic data from various cancer genomics resources.

### Tumor Immune Single-Cell Hub 2

Tumor Immune Single-cell Hub 2 (TISCH2, http://tisch.comp-genomics.org/home/) is a single‐cell RNA sequencing (scRNA‐seq) database focused on the tumor microenvironment that provides meta-information, cell type annotation, expression visualization, differential gene expression, Gene Set Enrichment Analysis (GSEA) results, transcription regulator analysis results, and cell-cell interaction results. We used TISCH2 to study the expression of TFF3 at the single-cell level.

### Breast Cancer Gene-Expression Miner v5.1

By using the Breast Cancer Gene-Expression Miner v5.1 (bc-GenExMiner v5.1) online dataset (http://bcgenex.centregauducheau.fr), the expression and prognostic value of TFF3 in BRCA were evaluated. The online dataset is a statistical mining tool that can perform statistical analysis of gene expression, correlation, and prognosis. It contains a large amount of published annotated genomic data as well as transcriptomic data from DNA microarrays and RNA-seq that have been annotated for BRCA. The information on this page was last updated on July 23, 2024, and it included 5023 RNA-seq patients and 11,552 DNA microarray patients. Using bc-GenExMiner v5.1, the correlation between TFF3 and the clinicopathological parameters of BRCA was assessed.

### Kaplan‒Meier Plotter

The Kaplan–Meier plotter (https://kmplot.com/analysis/) is capable of assessing the correlation between the expression of all genes (mRNA, miRNA, protein, and DNA) and survival in 40k+ samples from 21 tumor types, including breast, ovarian, lung, and gastric cancer. Sources for the databases include the Gene Expression Omnibus (GEO), the European Genome-phenome Archive (EGA), and the TCGA. We used KM plotter to explore the prognostic value of TFF3 in different immune cell subgroups in BRCA.

### Tumor IMmune Estimation Resource

The Tumor IMmune Estimation Resource (TIMER) (https://cistrome.shinyapps.io/timer/) can draw expression scatterplots between a pair of user-defined genes in a given cancer type, together with Spearman’s rho value and estimated statistical significance. We used TIMER to explore the correlation between TFF3 and immune cells in BRCA.

### TNMplot Database Analysis

The TNMplot tool (https://www.tnmplot.com/) was used to analyze differential gene expression in tumor tissues, normal tissues, and metastatic tissues. The TNM plot includes 56,938 unique multilevel quality-controlled samples from the GEO, GTEx, TCGA, and TARGET databases, along with 15,648 normal, 40,442 tumor, and 848 metastasis samples. With the use of this tool, the expression of TFF3 in normal, malignant, and metastatic tissues was compared and examined.

### LinkedOmics Database

We collected the genes co-expressed with TFF3 in BRCA from the LinkedOmics database (http://www.linkedomics.org/login.php) to perform gene set enrichment analysis. The signaling pathway associated with TFF3 in BRCA was analyzed for Kyoto Encyclopedia of Genes and Genomes (KEGG) enrichment using GSEA software and the ClusterProfiler package. Gene ontology (GO) enrichment and KEGG pathway analyses of co-expressed genes, depicted by the R package “ggplot2”, were performed via the R program “ClusterProfiler”.

### Immunohistochemistry Analysis

TFF3 protein expression was assessed in 149 BRCA tissues and 46 normal tissues via an anti-TFF3 antibody (ab108599; Abcam Inc., USA). Initially, formalin-fixed and paraffin-embedded (FFPE) tissues were deparaffinized and rehydrated. Next, antigen retrieval for TFF3 was performed in EDTA (1 mM, pH 8.0) buffer for 15 min in a microwave. The sections were then incubated with a rabbit anti-human TFF3 antibody (1:1200 dilution; Abcam Inc., USA) at 4 °C overnight. The FFPE samples were subsequently incubated with a biotinylated secondary antibody, and horseradish peroxidase (HRP)-conjugated goat anti-rabbit IgG (H + L) was used as the secondary antibody (1/500) at 37 °C for 30 min. This was followed by chromogen development with 3,3′-diaminobenzidine (DAB; Dako, Agilent, USA) and nuclear counterstaining with hematoxylin. Immunostaining reactivity was assessed via a light microscope (Olympus BX-53 with CCD DP74). Two independent, blinded pathologists, unaware of the clinicopathological data, evaluated the staining results. Discrepancies were resolved through consensus. TFF3 expression was semi-quantitatively analyzed in cancer cells and immune cells based on: Area of positivity (AP): 0 (0–5%), 1 (6%–25%), 2 (26%–50%), 3 (51%–75%), and 4 (> 75%); staining intensity (IS): 0 (negative), 1 (weak), 2 (moderate), and 3 (strong). The intensity distribution (ID) score was calculated as ID = AP × IS. X-Tile analysis revealed that the optimal cutoff for TFF3 expression was 4.8. The samples were classified as follows: high TFF3 expression (ID ≥ 4.5) or low TFF3 expression (ID < 4.5).

### Ethical Statement

This study was approved by the Ethics Committees of Maternal and Child Health Hospital of Hubei Province (No. HBMCH2023-K094), and the exemption of informed consent was in accordance with the Declaration of Helsinki. Because this was a retrospective study, informed consent was not needed. All the data/samples were fully anonymized before we accessed them.

### Statistical Analysis

All the statistical analyses were conducted via SPSS 22.0 software (USA) and R (V 3.6.3). The significance of TFF3 expression between tumor and normal tissues was determined via the Wilcoxon rank sum test. The relationships between TFF3 protein expression and associated clinicopathological variables were examined via the *χ*^2^ test or Fisher’s exact test. OS was estimated via the Kaplan–Meier method and the log-rank test. Univariate and multivariate Cox proportional hazard regression models were used to investigate the independent prognostic impacts on survival. Significance levels were set at ^*^P < 0.05, ^**^P < 0.01, and ^***^P < 0.001.

## Results

### Assessment of TFF3 mRNA Expression in BRCA via Multi-database Analysis

**Fig. 1:**
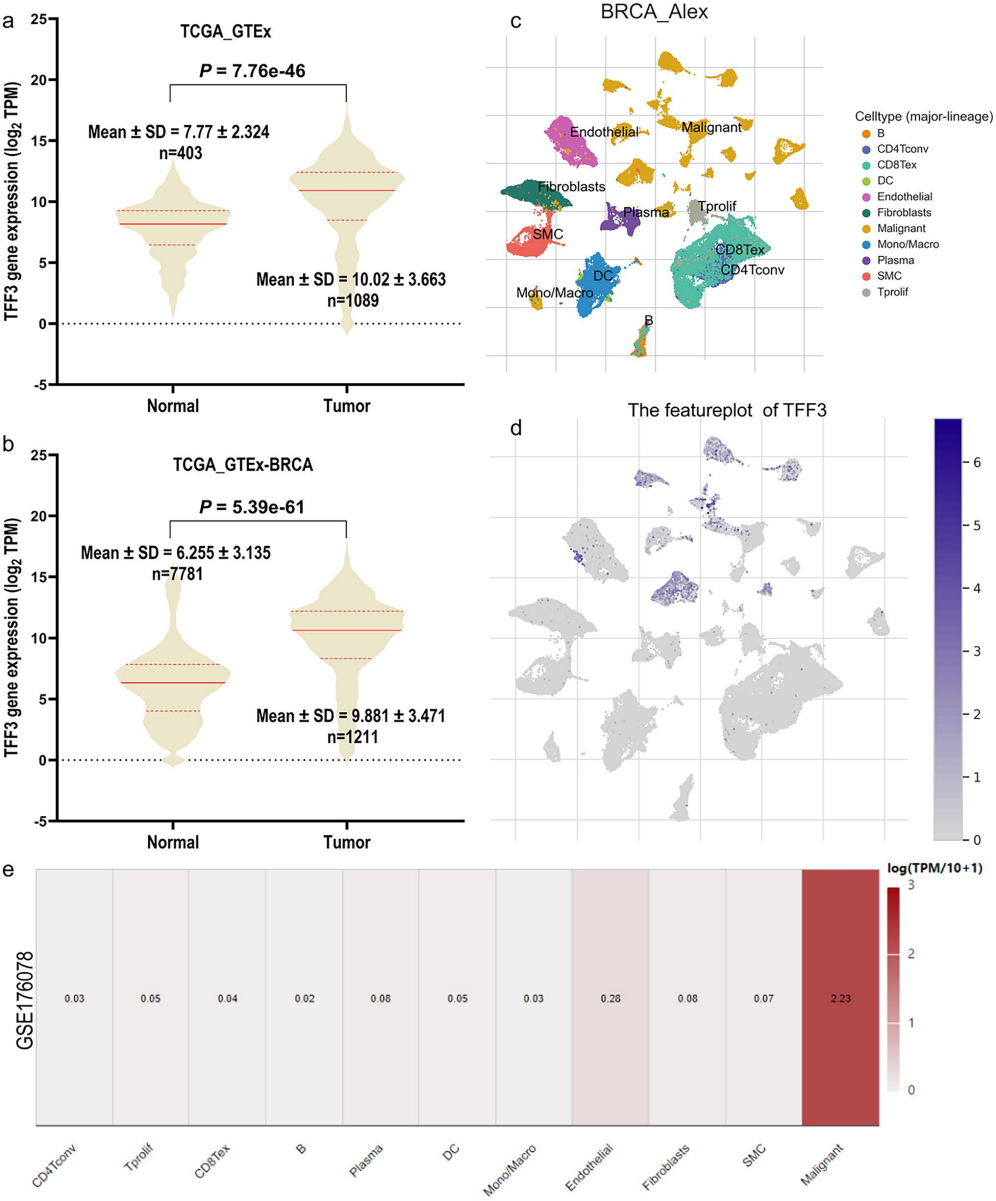
TFF3 expression in the transcriptome and single-cell transcriptome of BRCA patients. **a** Comparative analysis of TFF3 mRNA expression in BRCA tissues versus normal breast tissues from integrated TCGA and GTEx datasets; **b** TFF3 mRNA expression in BRCA tumor tissues versus normal breast tissues (excluding adjacent non-tumor samples) in TNMplot data; **c** scRNA-seq analysis reveals distinct cell subpopulations in BRCA; **d** feature plot illustrating TFF3 mRNA expression patterns at single-cell resolution; **e** cell type-specific TFF3 expression profile across malignant, endothelial, and immune cell populations

To evaluate TFF3 mRNA expression patterns in BRCA, we conducted a comprehensive analysis integrating data from four major databases: TCGA, GTEx, TNMplot, and TISCH2. For comparative analysis, we combined adjacent normal tissues from TCGA with normal breast tissues from GTEx to establish a robust normal tissue baseline (n = 7781) (Fig. 1). This integrated normal cohort demonstrated significantly lower TFF3 expression levels (6.255 ± 3.135) than did tumor tissues (9.881 ± 3.471, *n* = 1211) (*P* = 7.76e−46) (Fig. 1a). To further validate these findings, we analyzed TNMplot data after excluding potentially confounding adjacent samples from the normal group. This refined analysis confirmed markedly elevated TFF3 expression in tumor tissues (10.02 ± 3.663, n = 1089) compared with normal controls (7.77 ± 2.324) (*P* = 5.39e−61) (Fig. 1b). Single-cell resolution analysis through TISCH2 revealed the cellular distribution of TFF3 expression, which was predominant in malignant cells, endothelial cells, and monocytes/macrophages (Fig. 1c–e). This multi-platform approach consistently demonstrated significant upregulation of TFF3 in BRCA tumor tissues at both the bulk tissue and single-cell levels.

### Association of TFF3 mRNA Expression with Clinicopathological Features in BRCA

**Fig. 2:**
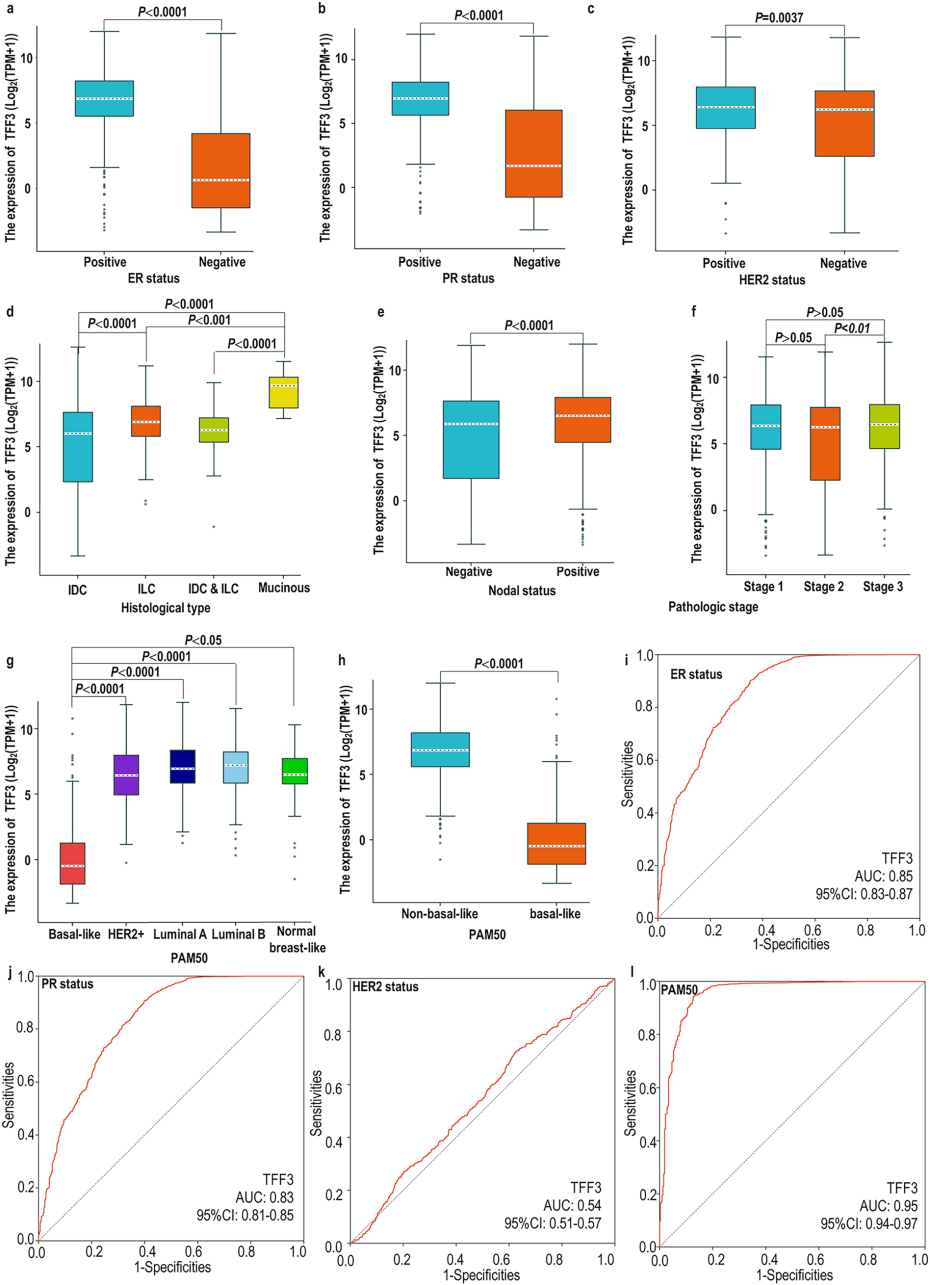
Association of TFF3 mRNA expression with clinicopathological features and diagnostic performance evaluation in BRCA. **a**–**c** TFF3 expression stratified by **a** ER status, **b** PR status, and **c** HER2 status. **d** Differential TFF3 expression across histological subtypes. **e** Correlation with lymph node involvement. **f** Association with pathological stage. **g**, **h** TFF3 expression patterns among PAM50 molecular subtypes. **i**–**l** ROC analysis evaluating the predictive value of TFF3 for **i** ER status (AUC = 0.85), **j** PR status (AUC = 0.83), **k** HER2 status (AUC = 0.54), and **l** basal-like versus nonbasal-like subtype discrimination (AUC = 0.95)

To elucidate the clinical relevance of TFF3 in BRCA, we evaluated its mRNA expression across key clinicopathological parameters, including hormone receptor status (ER, PR), HER2 amplification, PAM50 molecular subtypes, TNM staging, pathological stage, and histological classification (Fig. 2). Our analysis revealed that high expression of TFF3 was linked to ER, PR and HER2 (Fig. 2a–c). Notably, TFF3 mRNA expression was significantly correlated with histological type (Fig. 2d). We also found that TFF3 exhibited subtype-specific expression patterns within the PAM50 classification, with the highest levels in luminal tumors and the lowest in basal-like subtypes (Fig. 2d, e). Furthermore, a significant difference in the expression of TFF3 was observed only between the T1 and T2 stages (Fig. 2f–h). With respect to pathological stage, TFF3 expression was greater in stage 1 than in the other stages (Fig. 2i, j).

### Diagnostic Potential of TFF3 mRNA in BRCA Molecular Subtyping

To evaluate the diagnostic utility of TFF3 expression in the molecular subtyping of BRCA, we performed receiver operating characteristic (ROC) curve analysis. The ability of the variable TFF3 to predict ER-positive and negative outcomes was somewhat accurate (AUC = 0.85) (Fig. 2i). When BRCA was divided into PR-positive and PR-negative groups, the AUC was 0.83 (Fig. 2j). In predicting outcomes for the HER2-positive and HER2-negative groups, the variable TFF3 demonstrated lower accuracy, with an AUC of 0.54 (Fig. 2k). In predicting basal-like and nonbasal-like outcomes, the variable TFF3 had a certain level of predictive accuracy, with an AUC of 0.95 (Fig. 2l).

### TFF3 Overexpression as a Protective Factor in BRCA Prognosis

**Fig. 3:**
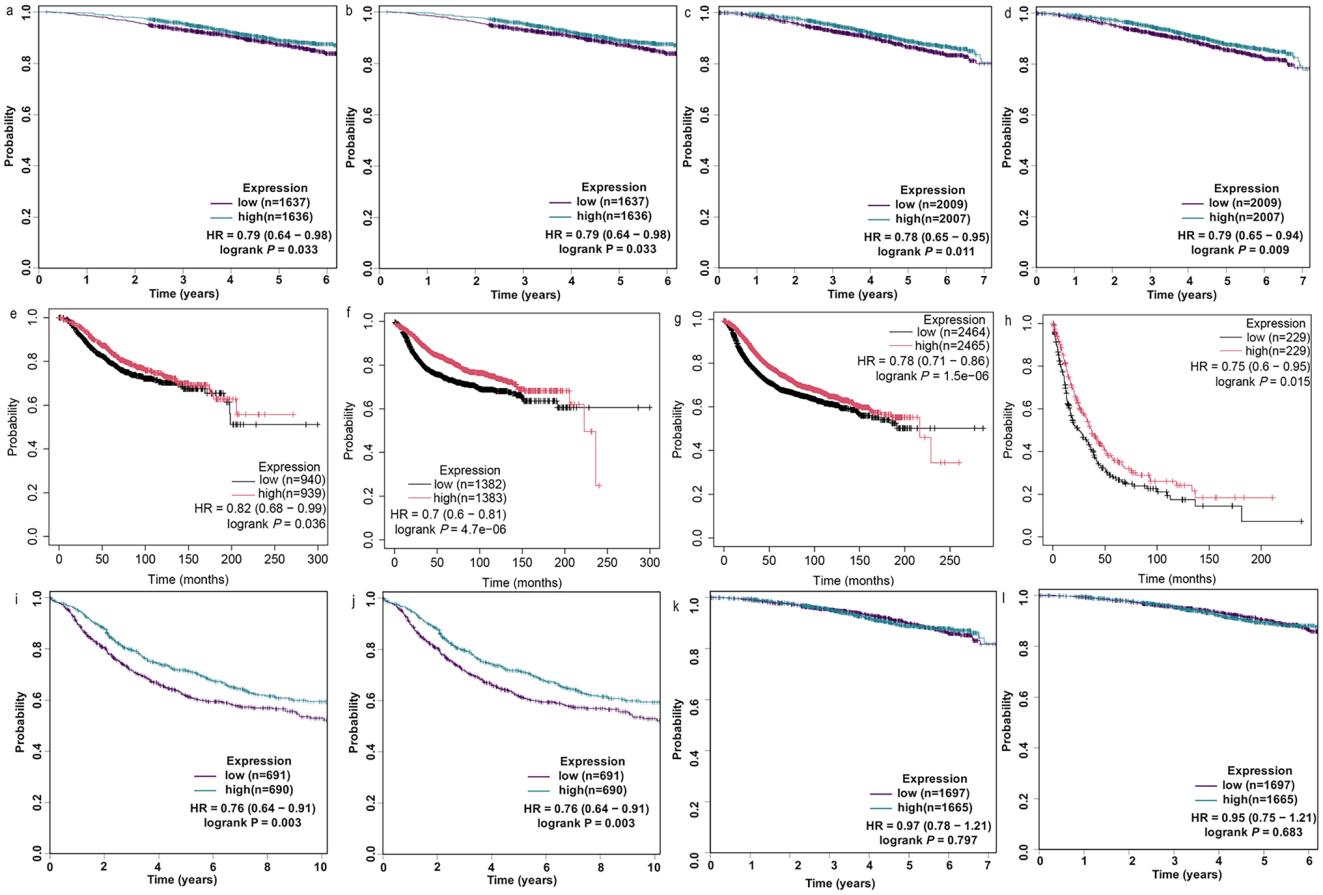
Prognostic value of TFF3 mRNA expression in BRCA across multiple databases. **a** OS in the SCAN-B cohort; **b** DFS in the SCAN-B cohort; **c** OS in the combined SCAN-B+TCGA cohort; **d** DFS in the combined SCAN-B+TCGA cohort; OS (**e**), DMFS (**f**), DFS (**g**) and RFS (**h**) in the KM plotter;**i** OS stratified by lymph node status in bc-GenExMiner v5.2 cohort; **j** DFS stratified by lymph node status in bc-GenExMiner v5.2 cohort; **k** OS stratified by ER status in bc-GenExMiner v5.2 cohort ; **l** OS stratified by PR status in bc-GenExMiner v5.2 cohort

To comprehensively evaluate the prognostic significance of TFF3 in BRCA, we performed multi-cohort analyses via the SCAN-B, SCAN-B+TCGA, KM plotter, and bc-GenExMiner v5.2 databases (Fig. 3). In SCAN-B, high expression of TFF3 was associated with better OS and DFS (Fig. 3a, b). These results were also validated in SCAN-B+TCGA (Fig. 3c, d). In the integrated gene chip data from the KM plotter, high expression of TFF3 was associated with better OS, DMFS, DFS and RFS (Fig. 3e–h), which included GSE1456, GSE16446, GSE16716, GSE20271, GSE20685, GSE20711, GSE22093, GSE3494, GSE37946, GSE42568, GSE45255, GSE48390, GSE58812, GSE65194, GSE69031, and GSE7390. In bc-GenExMiner v5.2, which studies lymph node-positive BRCA, we found that high expression of TFF3 is a protective factor (Fig. 3i, j). Our findings consistently demonstrated that elevated TFF3 expression serves as a protective factor across multiple clinical outcomes. Notably, this prognostic association was independent of ER/PR status (*P* ≥ 0.05) (Fig. 3k, l), suggesting that TFF3 may represent a novel biomarker beyond conventional hormone receptor stratification.

### Prognostic Significance of TFF3 mRNA across Clinical Subgroups in BRCA Patients

**Fig. 4:**
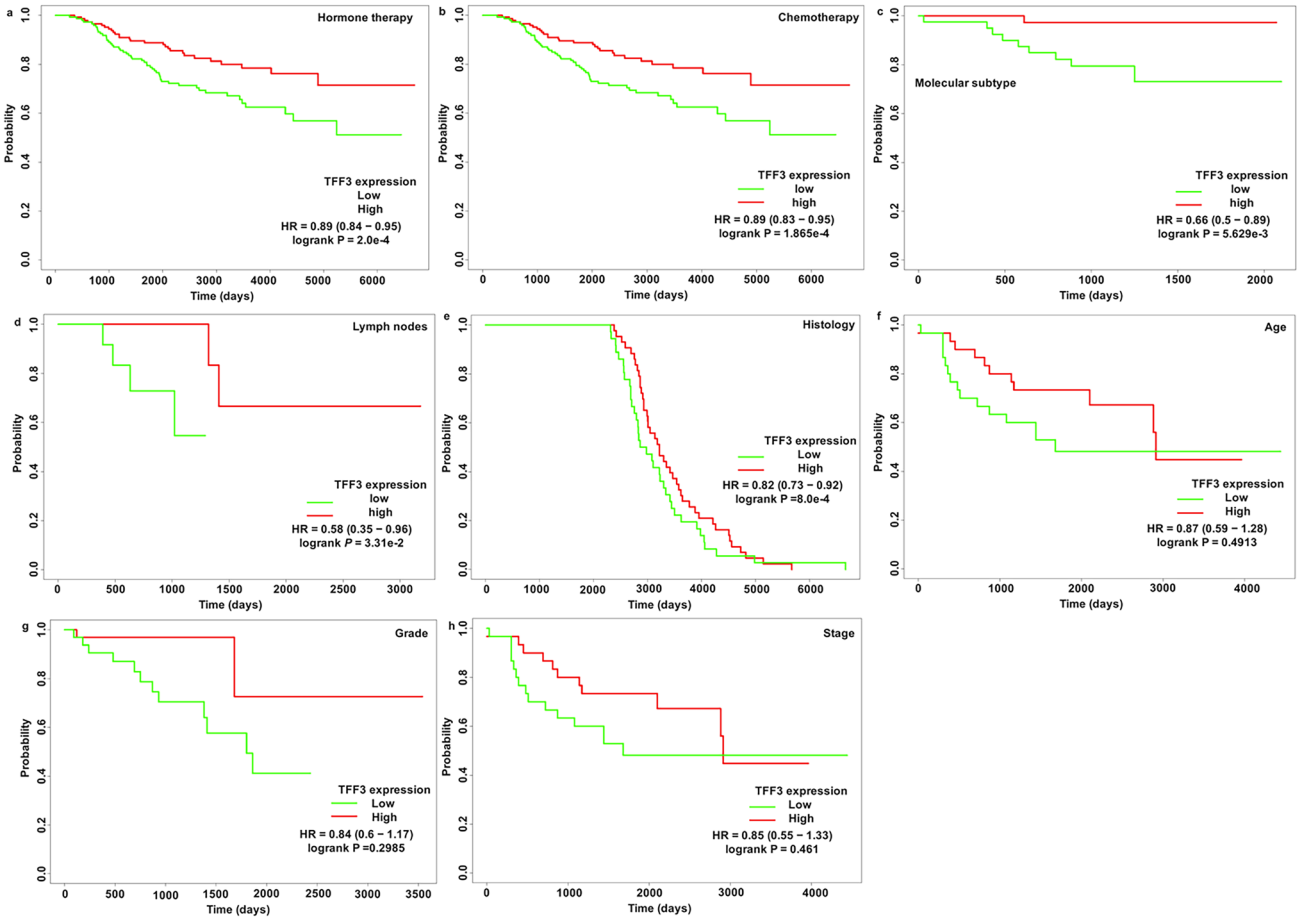
Prognostic value of TFF3 mRNA expression across clinical subgroups in patients with breast cancer (TCGA database). **a** Hormone therapy status; **b** chemotherapy treatment; **c** molecular subtypes; **d** lymph node involvement; **e** histological classification; **f** age subgroups; **g** tumor grade; **h** clinical stage

To evaluate the clinical relevance of TFF3 expression comprehensively, we conducted stratified survival analyses across key patient subgroups via TCGA data (Fig. 4). Our findings revealed that higher TFF3 expression levels were associated with improved survival outcomes in several stratified analyses, including patients receiving hormone therapy (HR = 0.89, 95% CI 0.84–0.95, *P* = 2.0 × 10^−4^) (Fig. 4a), those undergoing chemotherapy (HR = 0.89, 95% CI 0.83–0.95, *P* = 1.865 × 10^−4^) (Fig. 4b), different molecular subtypes (HR = 0.66, 95% CI 0.5–0.895, *P* = 5.629 × 10^−3^) (Fig. 4c), lymph node status subgroups (HR = 0.58, 95% CI 0.35–0.96, *P* = 3.31 × 10^−2^) (Fig. 4d), and distinct histological types (HR = 0.82, 95% CI 0.73–0.92, *P* = 8.0 × 10^−4^) (Fig. 4e). We also found that the expression level of TFF3 had no significant effect on patient age (HR = 0.87, 95% CI 0.59–1.28, *P* = 0.4913) (Fig. 4f), clinical grade (HR = 0.84, 95% CI 0.6–1.17, *P* = 0.2985) (Fig. 4g), and clinical stage (HR = 0.85, 95% CI 0.55–1.33, *P* = 0.461) (Fig. 4h). These results suggest that the prognostic value of TFF3 is particularly relevant in treatment-defined subgroups (hormone/chemotherapy recipients) and specific disease characteristics (molecular subtypes and lymph node status) but is independent of demographic factors or broad staging classifications.

### Association Between TFF3 mRNA Expression and the Tumor Immune Microenvironment

**Fig. 5:**
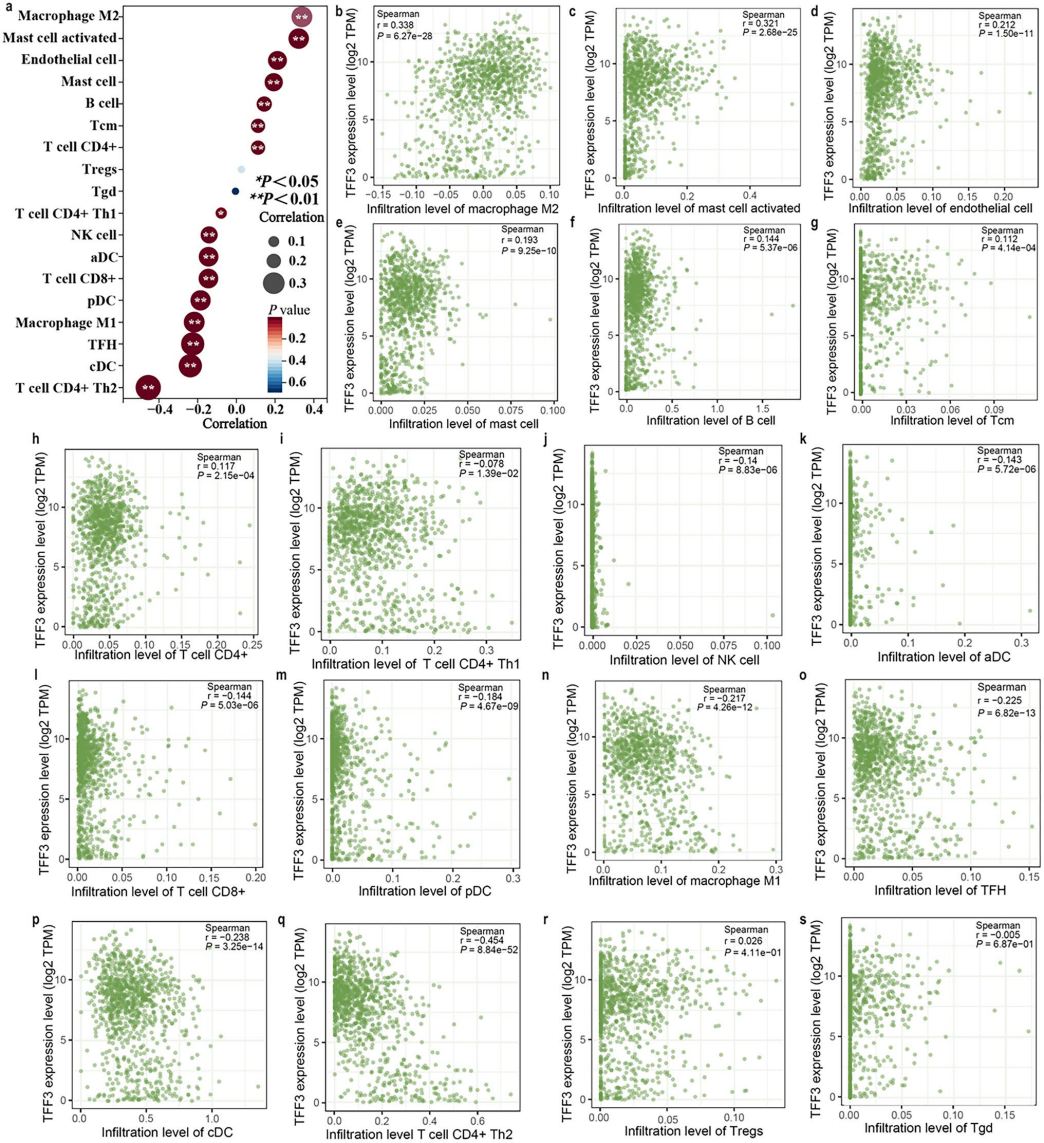
Correlation analysis between TFF3 mRNA expression and immune cell infiltration in BRCA. **a** Association between TFF3 expression and the overall immune infiltration score. **b**–**s** Specific correlations with individual immune cell subtypes: **b** M2 macrophages; **c** activated mast cells; **d** endothelial cells; **e** resting mast cells; **f** B cells; **g** CD4+ central memory T cells (Tcm); **h** CD4+ T cells; **i** CD4+ Th1 cells; **j** NK cells; **k** activated dendritic cells (aDCs); **l** CD8+ T cells; **m** plasmacytoid dendritic cells (pDCs); **n** M1 macrophages; **o** T follicular helper cells (TFHs); **p** conventional dendritic cells (cDCs); **q** CD4+ Th2 cells; **r** regulatory T cells (Tregs); **s** γδ T cells (Tγδ)

The composition of tumor-infiltrating immune cells significantly impacts cancer prognosis (Fig. 5). Using TCGA BRCA data, we systematically evaluated the correlations between TFF3 mRNA expression and immune cell infiltration patterns (Fig. 5a). TFF3 was positively associated with M2 macrophages, activated mast cells, endothelial cells, resting mast cells, B cells, CD4+ T cells,  and CD4+ central memory (Tcm) T cells (Fig. 5b–h). There was a negative association of TFF3 with CD4+ Th1 cells, NK cells, activated myeloid dendritic cells (aDCs), CD8+ T cells, plasmacytoid dendritic cells (pDCs), M1 macrophages, T follicular helper cells (TFHs), conventional dendritic cells (cDCs) and CD4+ Th2 cells (Fig. 5i–q). TFF3 was not negatively related with regulatory T cells (Treg) or gamma delta T cells (Tgd) (Fig. 5r, s). These findings position TFF3 as a potential immunomodulator in the breast tumor microenvironment, with particularly strong associations with immunosuppressive cell populations. This pattern suggests that TFF3 may contribute to an immune-tolerant microenvironment that could influence tumor progression and treatment response.

### TFF3 as a Potential Novel Immune Checkpoint in BRCA

**Fig. 6:**
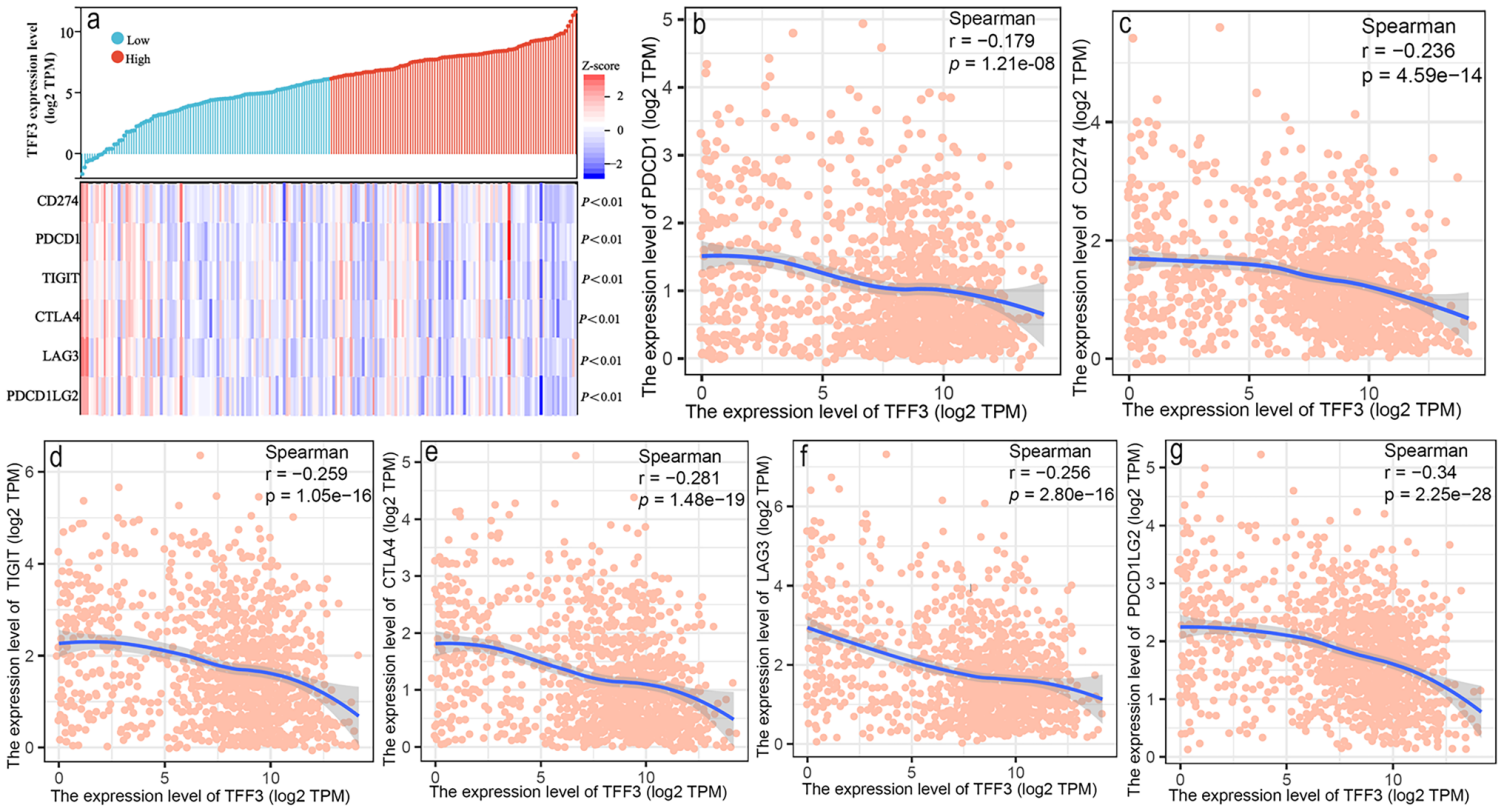
Correlation analysis between TFF3 expression and immune checkpoint molecules in breast cancer. **a** Heatmap visualization of TFF3 expression patterns relative to those of major immune checkpoint molecules. **b**‒**g** Scatter plots demonstrating significant negative correlations between TFF3 and **b** PD-1 (PDCD1), **c** PD-L1 (CD274), **d** TIGIT, **e** CTLA4, **f** LAG3, and **g** PD-L2 (PDCD1LG2) expression. All correlation analyses were performed via Spearman’s method

Given the emerging role of TFF3 as a putative immune regulator, we investigated its relationship with established immune checkpoint molecules (Fig. 6). Strikingly, our analysis revealed significant negative correlations between TFF3 expression and the expression of all major immune checkpoint markers analyzed (Fig. 6a), such as PDCD1, CD274, TIGIT, CTLA4, LAG3 and PDCD1LG2 (Fig. 6b–g). These consistent inverse relationships suggest that TFF3 may function as a novel class of immune modulator, potentially operating through mechanisms distinct from those of conventional checkpoint pathways. The strong negative correlations with multiple coinhibitory receptors (PD-1, CTLA-4, and LAG-3) and their ligands (PD-L1 and PD-L2) position TFF3 as a potentially important regulator in the BRCA immune microenvironment.

### Prognostic Significance of TFF3 mRNA Expression in Immune Cell-Specific Subgroups of BRCA

**Fig. 7:**
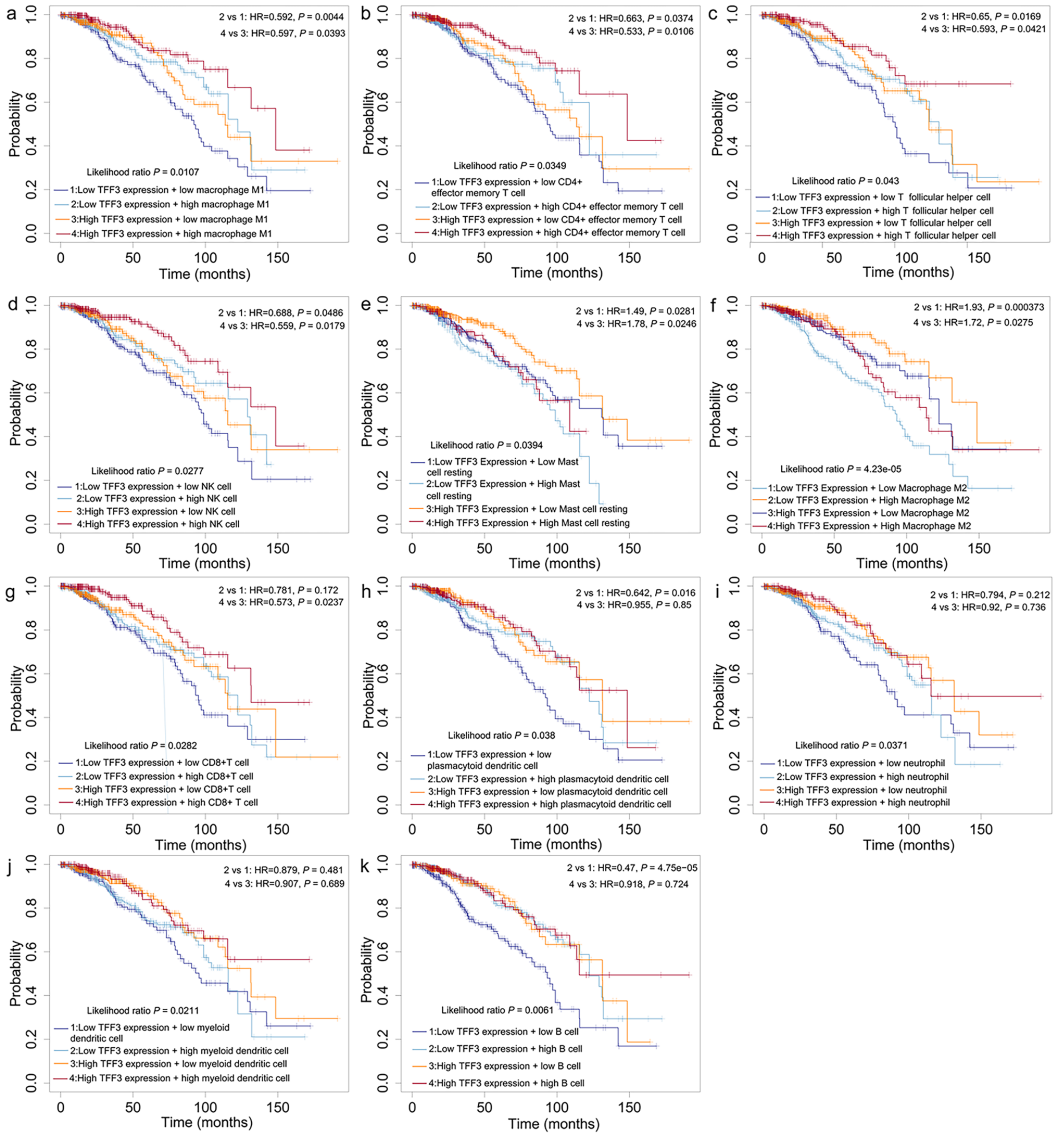
Prognostic value of TFF3 mRNA expression in immune cell-specific subgroups of BRCA patients. **a** M1 macrophage infiltration; **b** CD4+ effector memory T cells (Tem); **c** T follicular helper cells (TFHs); **d** Natural killer (NK) cells; **e** Resting mast cells; **f** M2 macrophage infiltration; **g** CD8+ cytotoxic T cells; **h** Plasmacytoid dendritic cells (pDCs); **i** Neutrophils; **j** Myeloid dendritic cells (mDCs); **k** B cells

Building upon previous findings demonstrating the association of TFF3 with immune cell infiltration, we investigated whether TFF3-mediated immunomodulation influences patient prognosis. The KM plotter database was used for Cox proportional hazards regression analysis (Fig. 7). Our findings suggested that the levels of TFF3 mRNA expression, especially elevated levels, were associated with improved prognosis in subsets with increased M1 macrophages (Fig. 7a), CD4+ effector memory T cells (Fig. 7b), T follicular helper cells (Fig. 7c), and NK cells (Fig. 7d). However, the level of TFF3 mRNA expression, especially elevated, was associated with poor prognosis in subsets with increased numbers of resting mast cells (Fig. 7e) and M2 macrophages (Fig. 7f). Elevated TFF3 mRNA expression was associated with improved prognosis in subsets with increased CD8+ T cells (Fig. 7g). Decreased TFF3 mRNA expression was associated with improved prognosis in subsets with increased numbers of plasmacytoid dendritic cells (Fig. 7h). The level of TFF3 mRNA expression was not associated with prognosis of breast cancer patients whose immune cell subgroups [such as subsets of neutrophils (Fig. 7i), myeloid dendritic cells (Fig. 7j) or B cells (Fig. 7k)] were enriched. These results suggest that the prognostic impact of TFF3 is highly dependent on the specific immune context, with particularly strong associations observed in T-cell- and macrophage-dominated microenvironments. Dichotomous effects (improved vs. worsened prognosis) which depend on the immune cell composition position TFF3 as a potential immunomodulatory target in BRCA therapy.

### Coexpression Network and Functional Enrichment Analysis of TFF3 in BRCA

**Fig. 8:**
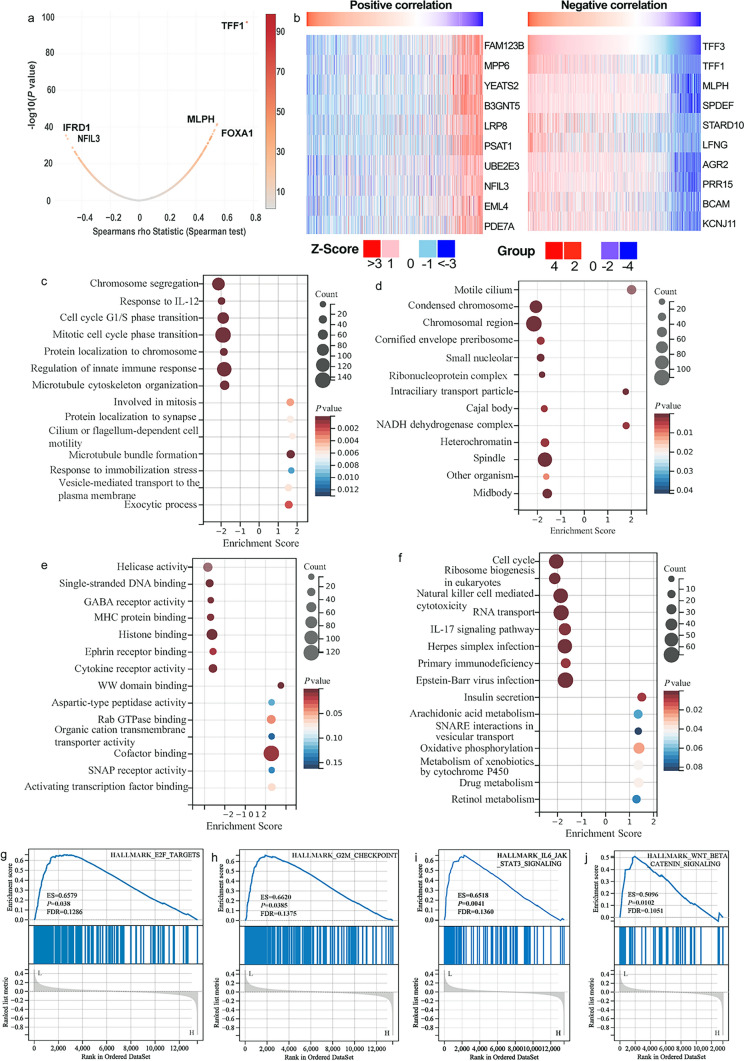
Coexpression network and functional characterization of TFF3 in BRCA. **a** Volcano plot showing genes significantly coexpressed with TFF3 (*P* < 0.05). **b** Heatmap visualization of the top 18 most strongly correlated genes (9 positively correlated genes and 9 negatively correlated genes). **c**–**e** GO enrichment analysis of TFF3-associated genes in **c** biological processes, **d** cellular components, and **e** molecular functions. **f** KEGG pathway enrichment analysis of TFF3 coexpressed genes. **g**–**j** Gene set enrichment analysis (GSEA) of hallmark pathways: **g** early estrogen response, **h** late estrogen response, **i** E2F targets, and **j** G2/M checkpoint

Using the LinkedOmics database, we performed comprehensive analyses to identify TFF3-associated genes and their functional implications in BRCA (Fig. 8). Spearman correlation analysis revealed genes correlated with TFF3 (Fig. 8a). The heatmap revealed 18 genes that were positively or negatively associated with TFF3 (Fig. 8b). GO and KEGG analyses revealed that these genes were involved in various biological processes and pathways, such as the regulation of the innate immune response, microtubule cytoskeleton organization involved in mitosis, spindle organization, protein localization to chromosomes, DNA replication, DNA conformation changes, the cell cycle G1/S phase transition, the mitotic cell cycle phase transition, the response to interleukin-12, and chromosome segregation (Fig. 8c). TFF3 was found to be associated with the replication fork, other organisms, spindle, heterochromatin, Cajal body, small nucleolar ribonucleoprotein complex, cornified envelope, preribosome, condensed chromosome, and chromosomal region in various cellular components and pathways (Fig. 8d). TFF3 was also found to be associated with cytokine receptor activity, double-stranded RNA binding, histone binding, Ran GTPase binding, MHC protein binding, GABA receptor activity, single-stranded DNA binding, hijacked molecular function, structural constituents of nuclear pores, and helicase activity in various molecular functions and pathways (Fig. 8e). The KEGG molecular pathways included primary immunodeficiency, Epstein‒Barr virus infection, the IL-17 signaling pathway, herpes simplex virus infection, graft-versus-host disease, natural killer cell-mediated cytotoxicity, glycosphingolipid biosynthesis, RNA transport, the cell cycle, and ribosome biogenesis in eukaryotes (Fig. 8f). This multi-dimensional analysis identified TFF3 as a potential regulator of critical cellular processes in BRCA, particularly in immune modulation, cell cycle control, and chromosomal maintenance. These diverse functional associations suggest that TFF3 may play pleiotropic roles in tumor biology.

### Gene Set Enrichment Analysis of TFF3-Related Signaling Pathways

To elucidate the biological functions of TFF3 in BRCA, we performed gene set enrichment analysis (GSEA) comparing gene expression profiles between the high- and low-TFF3 expression groups. The results indicated that TFF3 is involved in several biological processes and pathways, including the early estrogen response, late estrogen response, E2F-targets and the G2/M checkpoint (Fig. 8g–j). The normalized enrichment scores (NESs) and false discovery rates (FDRs) demonstrated robust associations of TFF3 with these fundamental biological processes, suggesting its potential role in hormone responsiveness and cell cycle progression in BRCA.

### Histological Validation of TFF3 Protein Expression in BRCA

**Fig. 9:**
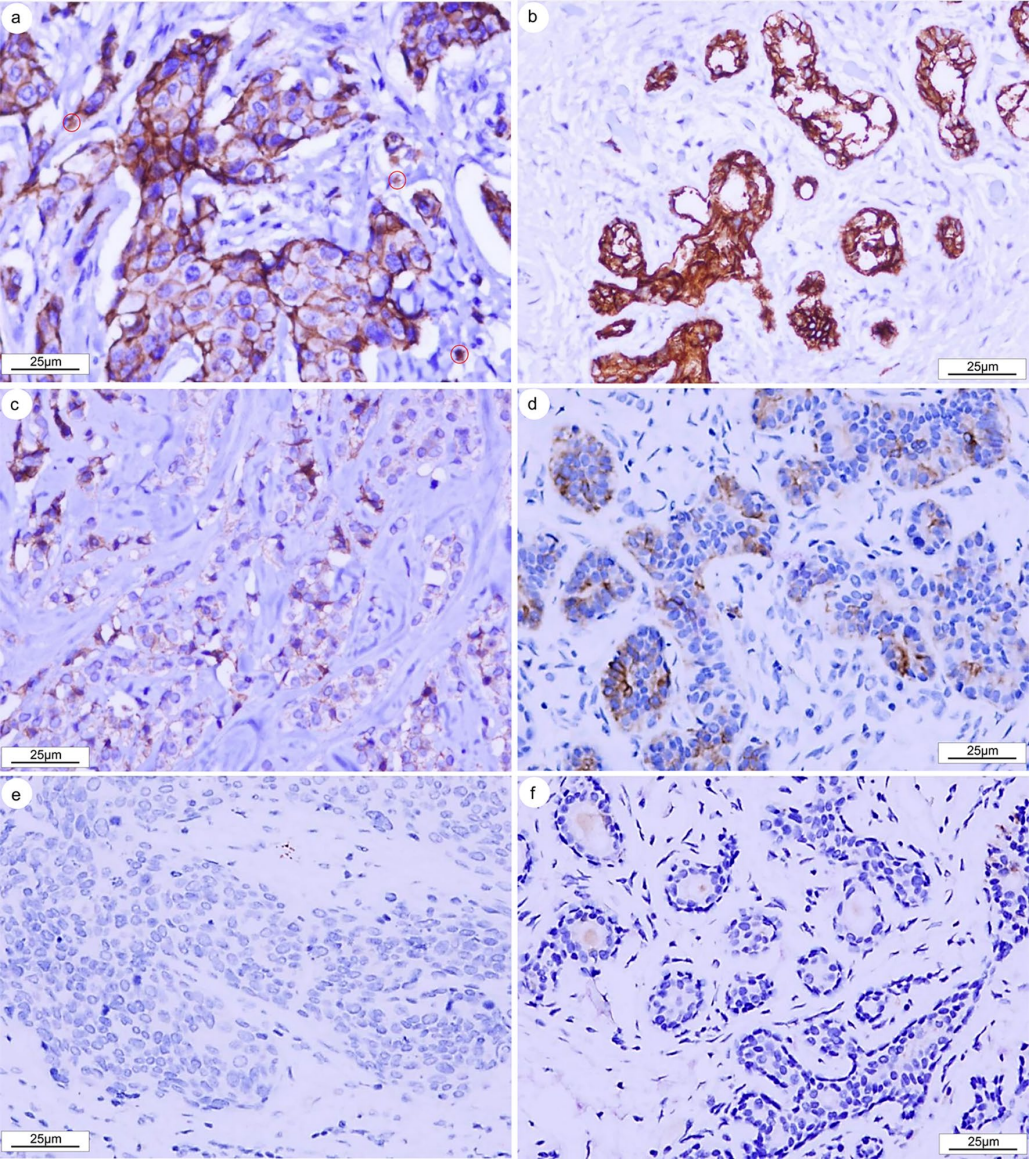
Immunohistochemical analysis of TFF3 protein expression in BRCA tissues. **a** Strong TFF3 immunoreactivity was observed in invasive ductal carcinoma cells (cytoplasmic and membranous staining) and tumor-associated immune cells (red circles indicate positive stromal macrophages). **b** Strong TFF3 staining in normal breast epithelium. **c** TFF3 focus weak expression in invasive ductal carcinoma. **d** Weak TFF3 positivity in normal breast glandular epithelium. **e** Negative TFF3 expression in invasive ductal carcinoma. **f** Absent TFF3 immunostaining in normal breast tissue. Scale bar: 25 μm

Immunohistochemical analysis revealed distinct patterns of TFF3 protein expression in BRCA specimens (Fig. 9). TFF3 protein exhibited differential expression between malignant breast tissues and paired adjacent normal tissues, with predominant localization in the cytoplasm and membranes of tumor cells (Fig. 9a–d). Strong immunoreactivity was detected in tumor-infiltrating immune cells, particularly stromal macrophages (indicated by red circles) (Fig. 9a). We noted that TFF3 expression was either absent or faint in some cancer cells or adjacent non-cancerous breast tissues (Fig. 9e, f). Moreover, high expression of TFF3 protein was observed in 107 out of 137 cancer tissues, which was significantly greater than the 11 out of 46 observed in corresponding non-cancerous breast tissues (*P* < 0.001). These results confirm TFF3 protein overexpression in BRCA and reveal its complex cellular distribution pattern, suggesting potential roles in both tumor cells and the tumor immune microenvironment. The frequent detection in malignant versus normal tissues supports its potential as a diagnostic biomarker.

### Clinicopathological Significance of TFF3 Protein Expression in BRCA

We evaluated the associations between TFF3 protein expression and clinicopathological characteristics in BRCA patients via the Chi-square test (Table 1). TFF3 expression in tumor cells was strongly correlated with invasive ductal carcinoma (IDC) pathological subtypes (*P* = 2.4896e−5) and PAM50 molecular subtypes (*P* = 0.5948e−3). Notably, TFF3 expression was significantly lower in basal-like tumors than in nonbasal-like tumors (*P* = 7.2085e−4). A distinct trend indicated higher TFF3 expression in the luminal A, luminal B, and HER2+ subtypes than in triple-negative BRCA (TNBC). Specifically, high TFF3 expression was detected in 59.2% (74/125) of nonbasal-like cases but only 8.33% (1/11) of basal-like BRCA cases. However, pairwise comparisons among the HER2+, luminal A, and luminal B subtypes did not reveal significant differences (*P* = 0.5895). No significant associations were detected between TFF3 expression and age, pathological grade, tumor size, lymph node status, or TNM stage (*P* > 0.05).

**Table 1:** Associations of the TFF3 protein with clinicopathological parameters of breast cancer tissues

Parameters	Cases	TFF3 protein in cancer cells		*P* value
		Low (%)	High (%)	
*Age (years)*				
≤ 45	14	6 (42.86)	8 (57.14)	0.8491
> 45	123	56 (45.53)	67 (54.47)	
*Pathological subtype*				
Invasive ductal carcinoma	116	25 (21.55)	91 (78.45)	2.4896e−5
Invasive lobular carcinoma	21	14 (66.67)	7 (33.33)	
*Pathological grade*				
1	23	11 (47.83)	12 (52.17)	0.2289
2	68	26 (38.24)	42 (61.76)	
3	46	25 (54.35)	21 (45.65)	
*Tumor size (mm)*				
≤ 30	121	54 (44.63)	67 (55.37)	0.685
> 30	16	8 (50.00)	8 (50.00)	
*Lymph node metastasis*				
Negative	86	40 (46.51)	46 (53.49)	0.7013
Positive	51	22 (43.14)	29 (56.86)	
*TNM stage*				
I	87	43 (49.43)	44 (50.57)	0.9280
II	37	17 (45.95)	20 (54.05)	
III	13	6 (46.15)	7 (53.85)	
*PAM50 molecular subtype*				
Basal-like	12	11 (91.67)	1 (8.33)	0.5948e−3
HER2+	11	6 (54.55)	5 (45.45)	
Luminal A	79	32 (40.51)	47 (59.49)	
Luminal B	35	13 (37.14)	22 (62.86)	

### Prognostic Significance of TFF3 Protein Expression in BRCA Patients

**Fig. 10:**
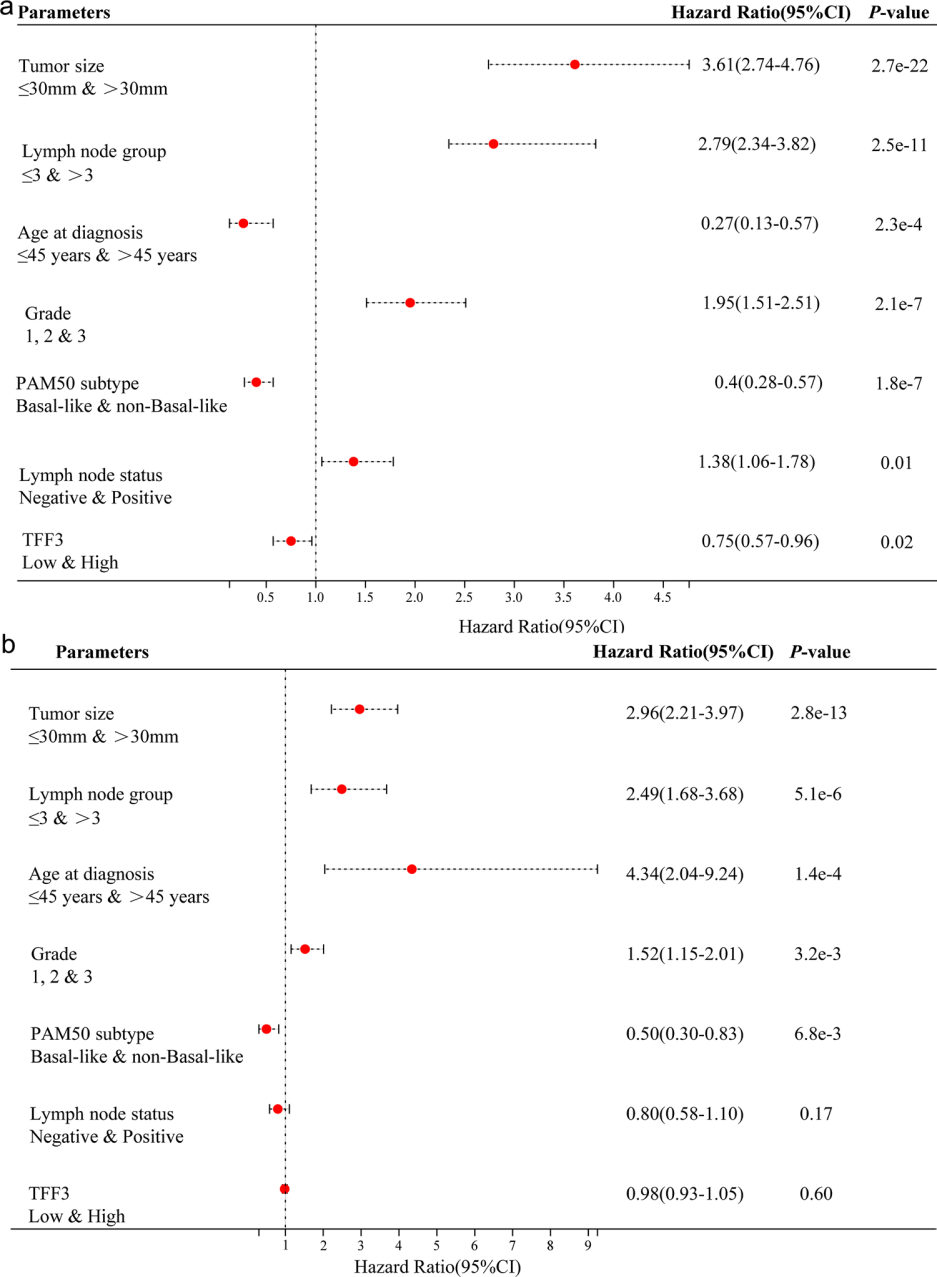
Prognostic value of TFF3 protein expression in breast cancer patients assessed via Cox proportional hazards regression models. **a** Univariate analysis of overall survival showing significant associations between TFF3 expression and clinicopathological factors. **b** Multivariate analysis demonstrating independent prognostic factors after adjustment for covariates. Hazard ratios (HRs) with 95% confidence intervals (CIs) and *P* values are indicated for each variable

We systematically evaluated the prognostic value of TFF3 protein expression along with other clinicopathological factors via Cox proportional hazards regression models (Fig. 10). According to the univariate analysis, there was a more favorable prognosis with high expression of TFF3 in tumor cells (*P* = 0.02; hazard ratio (HR): 0.75, 95% CI 0.57–0.96), patients older than 45 years at diagnosis (*P* = 2.3e−4; HR: 0.27, 95% CI 0.13–0.57) and patients with nonbasal-like PAM50 (*P* = 1.8e−7; HR: 0.4, 95% CI 0.28–0.57) (Fig. 10a). However, an unfavorable prognosis was associated with lymph node status (*P* = 0.01; HR: 1.38, 95% CI 1.06–1.78), metastatic lymph node number (*P* = 2.5e−11; HR: 2.79, 95% CI 2.34–3.82), tumor size (*P* = 2.7e−22; HR: 3.61, 95% CI 2.74–4.76), and pathological grade (*P* = 2.1e−7; HR: 1.95, 95% CI 1.51–2.51) (Fig. 10a). In the multivariate analysis, the overall model showed excellent prognostic discrimination (log-rank test = 2.173e−26; C-index = 0.6703). There was a more favorable prognosis only with nonbasal-like PAM50 (*P* = 6.8e−3; HR: 0.50, 95% CI 0.30–0.83) (Fig. 10b). There was also an unfavorable prognosis with respect to lymph node status (*P* = 0.01; HR: 1.38, 95% CI 1.06–1.78), metastatic lymph node number (*P* = 2.5e−11; HR: 2.79, 95% CI 2.34–3.82), tumor size (*P* = 2.7e−22; HR: 3.61, 95% CI 2.74–4.76), and pathological grade (*P* = 2.1e−7; HR: 1.95, 95% CI 1.51–2.51) (Fig. 10b).

## Discussion

The present study provides compelling evidence for the clinical and biological significance of TFF3 in BRCA through comprehensive multi-omics analyses via the TCGA, GTEx, TISCH2, and TNMplot databases. Our findings demonstrate that TFF3 is not merely a passive biomarker but also appears to play active roles in tumor biology, immune modulation, and treatment response. The consistency of the results across multiple independent cohorts and analytical platforms lends strong credibility to our conclusions.

We observed significant associations between TFF3 mRNA expression and key clinical parameters, including pathological subtype, clinical stage, molecular classification (luminal vs. basal-like), and hormone receptor status (ER/PR). ROC curve analysis indicated that TFF3 mRNA could serve as a potential biomarker for distinguishing basal-like subtypes, which are associated with better overall survival (OS) than triple-negative BRCA (TNBC) [24, 25].

To validate these findings at the protein level, we performed IHC on BRCA tissue. Our results demonstrated that the TFF3 protein was highly expressed in 78.1% (107/137) of the BRCA tissues compared with only 23.91% (11/46) in the adjacent normal tissues. IHC further confirmed TFF3 expression in both cancer cells and tumor-infiltrating immune cells. Notably, high TFF3 protein expression was significantly associated with luminal subtypes, lower histological grade, and positive ER/PR status [26]. High TFF3 expression is more frequently detected in invasive ductal carcinomas (IDCs) and low-grade tumors, reinforcing its association with less aggressive disease [27]. Survival analysis revealed a trend toward improved disease-free survival (DFS) in patients with high TFF3 expression, although this trend did not reach statistical significance in our cohort. However, analysis of the TCGA and METABRIC datasets confirmed that high TFF3 mRNA expression was significantly associated with better OS in luminal BRCA patients [28].

Previous studies have implicated TFF3 in various cancer-related pathways, including cell proliferation, apoptosis, and immune modulation, as demonstrated by GSEA [29]. Our GSEA results linking TFF3 to estrogen response pathways provide a mechanistic context for its luminal subtype preference and hormone therapy associations. The strong enrichment of genes related to cell cycle-related processes (E2F targets and the G2/M checkpoint) suggests that TFF3 may influence proliferative capacity, which is consistent with its increased expression in less aggressive luminal tumors. The diverse molecular functions identified (cytokine receptor activity, DNA binding) suggest pleiotropic roles that may explain the multifaceted clinical associations of TFF3. However, its function in cancer remains controversial—some studies suggest that TFF3 promotes tumor progression in colorectal and gastric cancers, whereas others indicate a tumor-suppressive role in certain contexts [30, 31].

Our immune landscape analysis revealed the complex involvement of TFF3 in shaping the breast tumor microenvironment. TFF3’s positive association with immunosuppressive M2 macrophages and negative correlation with cytotoxic immune populations (CD8+ T cells, NK cells) suggest that TFF3 may contribute to an immune-evasive microenvironment [26, 32]. Most intriguing is the consistent negative relationship with all major immune checkpoint molecules (PD-1, CTLA-4, LAG-3, and their ligands). This pattern differs fundamentally from known immune checkpoints and suggests that TFF3 may represent a novel class of immunoregulator, possibly functioning as a “checkpoint inhibitor”. The dichotomous prognostic associations depending on the immune context (favorable in M1/NK-rich environments vs. unfavorable in M2-rich environments) further underscore the complexity of the immunomodulatory role of TFF3. These findings contrast with studies in pancreatic and ovarian cancer, where TFF3 has been linked to chemoresistance and poor prognosis [33, 34]. This discrepancy highlights the context-dependent nature of TFF3 in cancer biology.

In the present study, TFF3 demonstrated considerable potential for the diagnosis and treatment of breast cancer. As a candidate biomarker, TFF3 assists clinicians in evaluating patient prognosis: it functions as a protective factor in the majority of breast cancer subtypes but is positively correlated with adverse outcomes in specific subtypes [25]. Additionally, TFF3 correlates with immune checkpoint molecules, facilitating the identification of patients who are likely to benefit from immunotherapy [20, 35]. As a therapeutic target, the modulation of TFF3 expression or activity may regulate the tumor immune microenvironment, laying a foundation for the development of novel therapeutic strategies. Nevertheless, further well-designed clinical studies are warranted to validate the clinical utility of TFF3 [36].

Our study demonstrated that TFF3 is upregulated in BRCA and may serve as a protective biomarker, particularly in luminal subtypes. Its association with favorable immune infiltration and improved survival suggests a potential immunomodulatory role. However, further mechanistic studies are needed to elucidate whether TFF3 directly influences immune responses or merely serves as a surrogate marker for less aggressive tumors. Given its differential roles across cancer types, TFF3 warrants further investigation as a potential therapeutic target or prognostic indicator in BRCA.

## Data Availability

The datasets presented in this study can be found in online repositories. The names of the repository/repositories and accession number(s) can be found in the article.
